# Nasal exposure to PM2.5 induces testicular injury and activation of pyroptosis-related signaling in rats: Inhibitor intervention and public transcriptomic analysis

**DOI:** 10.1371/journal.pone.0357773

**Published:** 2026-09-15

**Authors:** Yi Luo, Cao Wang, Mengxi Tian, Guangyou Lang, Kaixin Li, Zhongyan Zhang, Yu Chu, Qing Xiao, Ma Hong

**Affiliations:** Affiliated Hospital of Zunyi Medical University, Zunyi, Guizhou, China; University of Hyderabad, INDIA

## Abstract

**Objective:**

This study aimed to investigate the testicular injury induced by PM2.5 and the associated enhancement of NLRP3/caspase-1/GSDMD-related pyroptotic signaling in rats by integrating animal experiments with public transcriptomic analysis.

**Methods:**

Thirty-two male Sprague-Dawley rats were randomly assigned to four groups (n = 8 per group): a control group (saline), a caspase-1 inhibitor group (Z-YVAD-FMK, 5 mg/kg), a PM2.5 exposure group (10 mg/kg), and a combination group (PM2.5 plus Z-YVAD-FMK). The animals were treated for 4 weeks. The particle-size distribution of the prepared PM2.5 exposure suspension was characterized. Testicular histopathology was evaluated using hematoxylin and eosin (H&E) staining, and sperm count and morphology were assessed. Western blotting was employed to determine the expression levels of NLRP3, ASC, cleaved caspase-1, cleaved caspase-3, GSDMD-N, total caspase-1, and full-length GSDMD. Concurrently, the public GEO dataset GSE189187 (PM2.5-treated versus control GC-2spd cells; n = 3 per group) was analyzed for differential gene expression, functional enrichment, and protein-protein interaction (PPI) networks. Core modules and hub genes were identified using MCODE and cytoHubba in Cytoscape. Finally, the mRNA expression of Hmox1, Stat1, Irf9, and Usp18 in rat testicular tissue was measured using quantitative reverse-transcription PCR (qRT-PCR) to assess whether the candidate signals from the public transcriptomic dataset exhibited concordant changes in vivo.

**Results:**

Western blotting analysis revealed that exposure to PM2.5 significantly elevated the levels of several testicular proteins, including NLRP3, ASC, cleaved caspase-1, cleaved caspase-3, GSDMD-N, total caspase-1, and full-length GSDMD. Compared with PM2.5 alone, Z-YVAD-FMK co-treatment reduced ASC, GSDMD-N, cleaved caspase-1, total caspase-1, full-length GSDMD, and NLRP3, whereas cleaved caspase-3 did not differ significantly. These findings are consistent with partial attenuation of caspase-1/GSDMD-related signaling. The d(0.1), d(0.5), and d(0.9) values of the final exposure suspension were measured at 0.32 ± 0.04 μm, 1.15 ± 0.08 μm, and 2.38 ± 0.15 μm, respectively. In comparison to the control group, sperm counts decreased from 41.62 ± 3.38 × 10^6/mL to 11.48 ± 4.46 × 10^6/mL in the PM2.5 group, while the sperm abnormality rate increased from 6.31 ± 0.81% to 13.66 ± 1.87%. Following Z-YVAD-FMK treatment, sperm counts rose to 27.21 ± 3.34 × 10^6/mL, and the sperm abnormality rate decreased to 9.78 ± 0.72%. Public transcriptomic analysis identified a total of 217 differentially expressed genes (DEGs), comprising 91 upregulated and 126 downregulated genes. Functional enrichment and protein-protein interaction (PPI) network analyses suggested that PM2.5 treatment was linked to transcriptional alterations associated with interferon-mediated immune responses, inflammation, and cellular stress and injury, identifying Hmox1 and Stat1 as DEGs related to pyroptosis. Additionally, network topology analysis pinpointed STAT1, IRF9, members of the GBP family, IRGM1, and USP18 as potential hub nodes. Quantitative reverse transcription PCR (qRT-PCR) demonstrated increased Hmox1 mRNA expression alongside decreased Stat1, Irf9, and Usp18 mRNA expression in the testes of PM2.5-exposed rats. These alterations were partially reversed by Z-YVAD-FMK; because the public dataset and animal experiment differed in species and sample type, this comparison was interpreted as exploratory rather than as direct validation.

**Conclusion:**

PM2.5 exposure induced histopathological injury in rat testes and was accompanied by enhanced NLRP3/caspase-1/GSDMD-related signaling. Z-YVAD-FMK partially alleviated tissue injury and reduced several pyroptosis-related proteins, whereas cleaved caspase-3 did not differ significantly between the PM2.5 and PM2.5 plus Z-YVAD-FMK groups. The rat-tissue qRT-PCR findings provided a cross-system comparison for selected candidate signals identified in the public transcriptomic dataset, rather than direct mechanistic validation. The transcriptomic analysis further suggested immune-inflammatory and stress-related remodeling in GC-2spd cells; the resulting STAT/IRF-GBP-IRGM-USP18/PARP network should be interpreted as exploratory.

## 1 Introduction

### 1.1 PM2.5 and male reproductive toxicity

Male infertility has emerged as a significant global health concern, with impaired sperm quality being a key contributing factor [1]. As air pollution has intensified, the association between PM2.5 and male reproductive health has garnered increasing attention. PM2.5 is a complex mixture that possesses substantial toxic and pathogenic potential. Epidemiological evidence indicates that long-term exposure to air pollution correlates with an elevated risk of male infertility [2], and multicenter population-based studies have further linked exposure to PM2.5 and its constituents with reduced semen quality [4,5]. PM2.5 may contribute to male reproductive toxicity by disrupting the testicular immune microenvironment, inducing inflammatory responses, and interfering with spermatogenesis [3]. Animal studies have demonstrated that PM2.5 damages the male reproductive system by inducing redox imbalance, ferroptosis, and impairing testosterone synthesis [6,7]. Furthermore, it may induce ferroptosis in spermatocytes through iron overload and disruption of redox homeostasis [8]. Exposure to air pollution at different stages of spermatogenesis may also impair semen quality, potentially by altering seminal plasma metabolism [27,28]. Therefore, elucidating the toxicological mechanisms underlying PM2.5-induced injury to the male reproductive system is of considerable importance.

### 1.2 PM2.5 and pyroptosis

Pyroptosis is an inflammasome-driven form of programmed inflammatory cell death. In the canonical pathway, NLRP3 and ASC recruit and activate caspase-1, which subsequently cleaves GSDMD to form membrane pores and promotes the maturation and release of IL-1β and IL-18, leading to cell swelling, lysis, and amplification of inflammation [10,22]. Various environmental pollutants can activate the NLRP3 inflammasome through oxidative stress and inflammatory signaling, thereby inducing pyroptosis [11]. PM2.5 promotes NLRP3 inflammasome activation and IL-1β release, suggesting that the NLRP3/caspase-1 axis may serve as an important mediator of PM2.5-induced inflammatory injury [12]. Furthermore, PM2.5-induced ferroptosis and inflammasome activation may also reinforce each other [13]. Previous studies in respiratory models have demonstrated that PM2.5 can activate the NLRP3 inflammasome and trigger pyroptosis through upstream pathways such as ROS/NF-κB signaling. However, direct experimental evidence showing that PM2.5 induces testicular cell pyroptosis via the NLRP3/caspase-1 pathway remains limited [9]

### 1.3 PM2.5 and public transcriptomic analysis

With advances in high-throughput sequencing, proteomics, and public database resources, systems biology and bioinformatics approaches have been widely employed to identify key genes, protein biomarkers, and molecular pathways associated with complex diseases. Systematic reviews of proteomic studies can reveal shared protein biomarkers involved in the pathogenesis of respiratory diseases [24]. Additionally, systems biology analyses of public datasets can elucidate key genes and molecular pathways implicated in the progression from monoclonal gammopathy of undetermined significance to multiple myeloma [25]. Furthermore, computational approaches can pinpoint key genes and biological pathways in chronic lung diseases [26]. Collectively, these studies suggest that integrating public omics data with differential expression, functional enrichment, and protein-protein interaction (PPI) network analyses can enhance the identification of candidate molecular networks underlying complex diseases and environmental exposure-related injuries. Public omics databases and bioinformatics analyses have also been utilized in PM2.5 toxicology to identify potential pathways and regulatory genes [15], as well as to investigate disease patterns and mechanisms associated with PM2.5 exposure [14]. The GSE189187 dataset used in the present study was generated from a metabolomic and transcriptomic investigation of PM2.5-exposed mice and GC-2spd cells [16].

In the present study, we established a rat model of intranasal PM2.5 exposure, with or without Z-YVAD-FMK treatment, and reanalyzed transcriptomic data from PM2.5-treated GC-2spd cells in GSE189187. Differential expression, functional enrichment, and exploratory PPI network analyses were used to identify candidate genes and immune-inflammatory modules. The mRNA expression levels of Hmox1, Stat1, Irf9, and Usp18 were subsequently measured in rat testicular tissue using qRT-PCR to compare the in vivo expression patterns of selected candidate signals with the public cell-line dataset. By integrating testicular histopathology, sperm parameters, caspase-1/GSDMD-related protein expression, public transcriptomic analysis, and tissue-level qRT-PCR measurements, this study aimed to explore potential immune-inflammatory mechanisms associated with PM2.5-induced male reproductive injury.

## 2 Materials and methods

### 2.1 Source of PM2.5, suspension preparation, and available physicochemical information

The PM2.5 utilized in this study was NIST Standard Reference Material (SRM) 1648a Urban Particulate Matter, procured from Dongguan Baishun Biotechnology Co., Ltd. (catalog no. 1648a; 2 g; NIST). SRM 1648a serves as a standard reference material for urban atmospheric particulate matter and encompasses both inorganic constituents and various organic pollutants. According to the NIST SRM 1648a certificate, the volume-based particle-size distribution, measured by laser diffraction under specified aqueous dispersion conditions, yielded d(0.1) = 1.35 μm, d(0.5) = 5.85 μm, and d(0.9) = 30.1 μm. Prior to usage, an appropriate amount of PM2.5 was suspended in sterile saline within 24 hours and agitated overnight at 60 rpm in a 37°C incubator shaker to achieve the required concentration. The resulting suspension was stored at 4°C and thoroughly mixed before each administration to minimize particle sedimentation and aggregation. The administration volume was adjusted based on body weight to ensure a PM2.5 dose of 10 mg/kg. To characterize the PM2.5 saline suspension intended for intranasal exposure, particle-size analysis was conducted on three independently prepared batches under the same medium, concentration, and preparation conditions employed for animal administration. The d(0.1), d(0.5), and d(0.9) values of the final exposure suspension were 0.32 ± 0.04 μm, 1.15 ± 0.08 μm, and 2.38 ± 0.15 μm, respectively. The span of the particle-size distribution was 1.79, indicating that the suspension predominantly comprised micrometer-scale particles with a moderately broad size distribution.

### 2.2 Experimental animals, grouping, and PM2.5 exposure protocol

Experimental animals were obtained from the Laboratory Animal Center of Zunyi Medical University (license no. SYXK [Guizhou] 2021−0004). The study received approval from the institutional animal welfare and ethics committee (approval no. zyfy-an-2023–0013). Thirty-two specific-pathogen-free male Sprague-Dawley rats (4 weeks old; 95 ± 10 g) were housed individually in a barrier facility. The rats were randomly assigned to four groups (n = 8 per group): saline control (intranasal instillation); Z-YVAD-FMK (intraperitoneal injection, 5 mg/kg); PM2.5 (intranasal instillation, 10 mg/kg); and PM2.5 plus Z-YVAD-FMK (intranasal PM2.5 followed 30 minutes later by intraperitoneal Z-YVAD-FMK). Z-YVAD-FMK was utilized as a caspase-1 inhibitor for intervention-based validation. The animals were treated for five consecutive days, followed by two days without treatment, and this cycle was repeated for four weeks.

### 2.3 Animal-welfare monitoring, ethical procedures, and euthanasia

Throughout the experiment, the rats were monitored daily for their general condition, activity levels, food and water intake, respiration, coat appearance, and changes in body weight. Humane endpoints included persistent body weight loss exceeding 20%, marked respiratory distress, sustained refusal of food, a substantial reduction in activity, or signs of a moribund state. Animals meeting any of these criteria were to be withdrawn from the study and euthanized. At the end of the experiment, euthanasia was performed using gradual-fill CO2 inhalation, and death was confirmed by the cessation of respiration and heartbeat, as well as the absence of the pupillary reflex. Administration and tissue collection procedures were completed as efficiently as possible to minimize pain and stress.

### 2.4 Body-weight measurement, organ-index calculation, and sperm analysis

After 4 weeks of exposure, the rats were weighed and euthanized, and both the testes and epididymides were promptly excised. The testes were weighed, and the testicular organ index was calculated using the following formula: organ index = wet organ weight (g) / body weight (g) × 100%. One testis was fixed for H&E staining, while the contralateral testis was divided and frozen for Western blotting and qRT-PCR. The cauda epididymis was utilized for sperm counting and morphological assessment. Fresh epididymides were separated from the testes and transferred to 2 mL of prewarmed phosphate-buffered saline (PBS). The epididymides were cut into small pieces and incubated at 37°C for 20 minutes to facilitate sperm release. A 10-μL aliquot of the sperm suspension was diluted to 10 mL with PBS, loaded into a Neubauer hemocytometer, and counted under a light microscope, with at least 2,000 sperm examined. For morphological assessment, 10 μL of the sperm suspension was diluted with 190 μL of PBS, and 10 μL of the diluted sample was smeared onto a clean glass slide. After air-drying for 10 minutes, the smear was fixed in 4% formaldehyde for 20 minutes, stained, and imaged under a light microscope. At least 200 sperm were counted in randomly selected fields on each slide. The sperm abnormality rate was defined as the proportion of sperm exhibiting head, neck, or tail abnormalities, including missing, shortened, or coiled structures, among all sperm counted.

### 2.5 Testicular H&E staining and histopathological evaluation

Testicular tissues were rinsed with PBS, fixed in 4% paraformaldehyde for 24 hours, dehydrated, embedded in paraffin, sectioned, deparaffinized, stained with hematoxylin and eosin (H&E), dehydrated again, and mounted. The architecture of seminiferous tubules, organization of germ cells, and vacuolar changes were evaluated using a light microscope (Leica DM3000, Germany). Four fields were randomly selected from each animal, and all images were acquired at the same magnification using identical imaging parameters.

### 2.6 Western blotting and qRT-PCR

Testicular proteins were quantified using the bicinchoninic acid assay. The samples were separated by sodium dodecyl sulfate-polyacrylamide gel electrophoresis and subsequently transferred to polyvinylidene difluoride membranes. Following blocking, the membranes were incubated with primary antibodies for 16 hours at 4°C with gentle agitation. The primary antibodies targeted NLRP3 (ET1610−93, HUABIO), ASC (R381245, ZENBIO), caspase-1 (341030, ZENBIO), GSDMD (ER1901−37, HUABIO), cleaved caspase-3 (ET1608−64, HUABIO), cleaved caspase-1 (ER1905−47, HUABIO), GSDMD-N (HA721144, HUABIO), and β-actin (200068-6D7, ZENBIO), the latter of which served as the loading control. β-Actin was diluted 1:10,000, while all other primary antibodies were diluted 1:800. The membranes were washed three times with Tris-buffered saline containing Tween 20 and incubated for 2 hours at room temperature with rabbit IgG secondary antibody (511203, ZENBIO; 1:1,000). Immunoreactive bands were detected using a Tanon imaging system (Tanon Life Science, Shanghai, China). Band intensities were quantified in ImageJ and normalized to β-actin. Uncropped original Western blot images were provided as supporting information.

Frozen testicular tissue was collected from all groups, with eight animals per group serving as independent biological replicates. Total RNA was extracted using the FastPure Cell Total RNA Isolation Kit (Vazyme, RC101). The quality and concentration of RNA were assessed using an ultramicro nucleic-acid/protein analyzer (Implen, Germany). RNA was reverse-transcribed into cDNA using HiScript III RT SuperMix for qPCR (Vazyme, R323), and amplification was performed with 2 × ChamQ Universal SYBR qPCR Master Mix (Vazyme, Q711). Three technical replicates were analyzed for each biological sample. The cycling conditions were as follows: initial denaturation at 95°C for 5 minutes; 40 cycles of denaturation at 95°C for 15 seconds, annealing at 60°C for 30 seconds, and extension at 72°C for 30 seconds; followed by melting-curve analysis. GAPDH was employed as the reference gene. Primers were designed by Guizhou Keaode Biotechnology Co., Ltd., and the relative mRNA expression levels of Hmox1, Stat1, Irf9, and Usp18 were calculated using the 2^-ΔΔCt method. Primer sequences are listed in Table 1.

**Table 1:** Primers used for quantitative reverse-transcription PCR.

Gene	Forward primer (5′-3′)	Reverse primer (5′-3′)
Hmox1	TTTTCACCTTCCCGAGCATC	ATCTCCAGAGTGTTCATGCG
Stat1	GAAAAGCAAGCGTAATCTCCAGG	TTCCTCTCCTCCTTCAGACAGTTG
Irf9	AGGACCCAGTGTTCATGGA	GGTGAGCAGCAGCGAGTAGT
Usp18	TGGCTTGGCTGTAGTTTGTCTCCCA	TCCCACTGGGCCCTGTCCCTT
GAPDH	AAGTTCAACGGCACAGTCAAGC	CATACTCAGCACCAGCATCACC

### 2.7 Dataset selection

The GSE189187 dataset was retrieved from the Gene Expression Omnibus using the keywords “PM2.5” and “GC-2spd cells.” This dataset comprises mRNA expression profiles from six samples of the mouse spermatocyte-derived GC-2spd cell line, including three control samples and three samples treated with PM2.5. GC-2spd cells were exposed in vitro to 100 μg/mL PM2.5 for 48 hours prior to transcriptome sequencing. The PM2.5 utilized in the original study was NIST SRM 1648a urban particulate matter, suspended in PBS and sonicated before cellular exposure [16].

### 2.8 Dataset processing and statistical analysis

**Fig 1:**
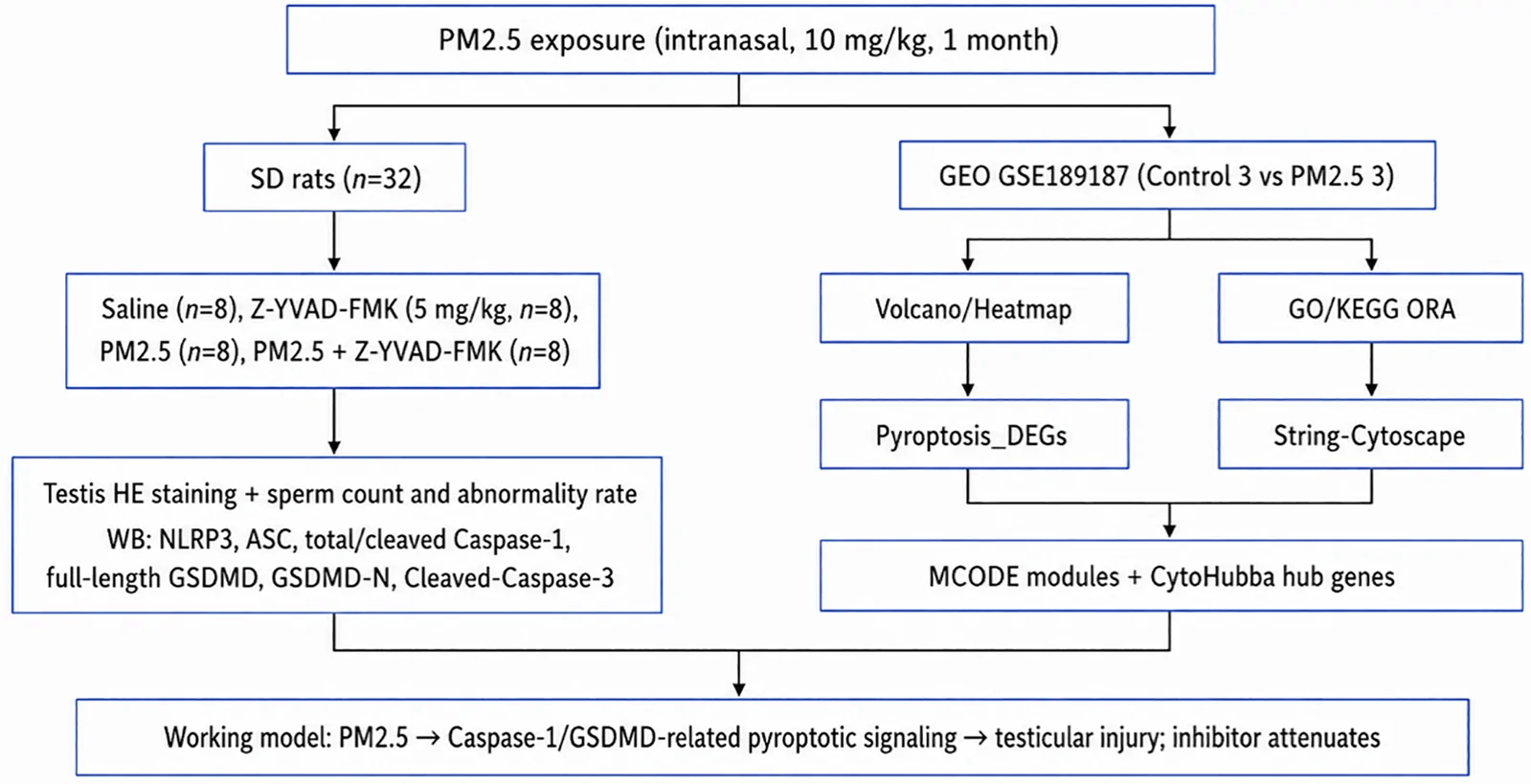
Study design and analytical workflow.

GSE189187 (PM2.5-treated versus control GC-2spd cells; n = 3 per group) was analyzed to identify differentially expressed genes (DEGs), visualize highly ranked genes in a heatmap, perform over-representation analysis (ORA), and construct protein-protein interaction (PPI) networks (Fig 1). Bioinformatics analyses comparing the PM2.5 and control groups were conducted in RStudio using the relevant R packages. Due to the small sample size, false discovery rate adjustment was expected to be conservative. Therefore, candidate genes were screened using a combined threshold of P.Value and |log2FC|, while adjusted P values were retained in the output tables as a reference for robustness. Gene Ontology (GO) and Kyoto Encyclopedia of Genes and Genomes (KEGG) enrichment analyses were performed separately for the upregulated and downregulated screening sets. A pyroptosis-related gene set was compiled from mechanistic reviews and related literature and intersected with DEG_screen_all to obtain Pyroptosis_DEGs [10,22]. An expanded 32-gene pyroptosis/interferon candidate set was submitted to STRING to construct an exploratory PPI network at a confidence threshold of 0.7; the exact input list and DEG-mapping status are provided in S2 File. The network was imported into Cytoscape; MCODE was used to identify modules, and cytoHubba was used to rank the top 10 nodes [17–20]. Because the expanded network was not restricted to the 217 screened DEGs, network topology was treated as hypothesis-generating. Continuous data are presented as the mean ± standard deviation. Animal data were analyzed using GraphPad Prism 9.0. Normality was assessed using the Shapiro-Wilk test, and homogeneity of variances was evaluated using Levene's test. When both assumptions were satisfied, multiple-group comparisons were performed using one-way analysis of variance followed by Tukey's test for pairwise comparisons. A P value of < 0.05 was considered statistically significant.

## 3 Results

### 3.1 PM2.5 exposure alters gene expression in GC-2spd cells

After normalizing the GSE189187 data, the volcano plot revealed a distinct distribution of differentially expressed genes between the PM2.5-treated and control groups (Fig 4A). Using a P-value threshold of < 0.05 and |log2FC| > 0.585 as screening criteria, a total of 217 differentially expressed genes (DEGs) were identified, comprising 91 upregulated and 126 downregulated genes.

**Fig 2:**
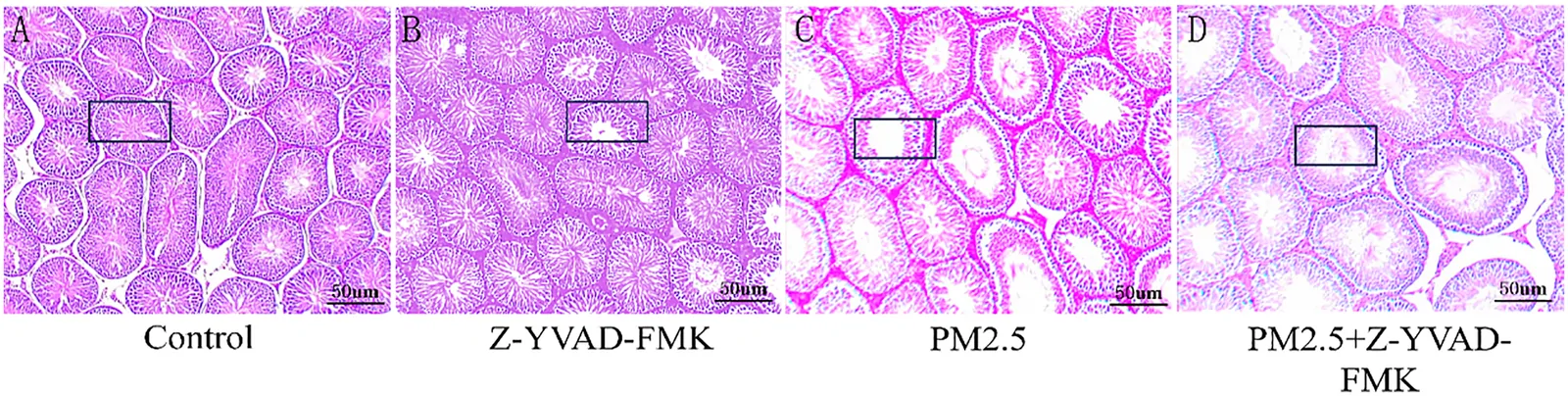
Histopathological changes in rat testicular tissue following PM2.5 exposure.

**Fig 3:**
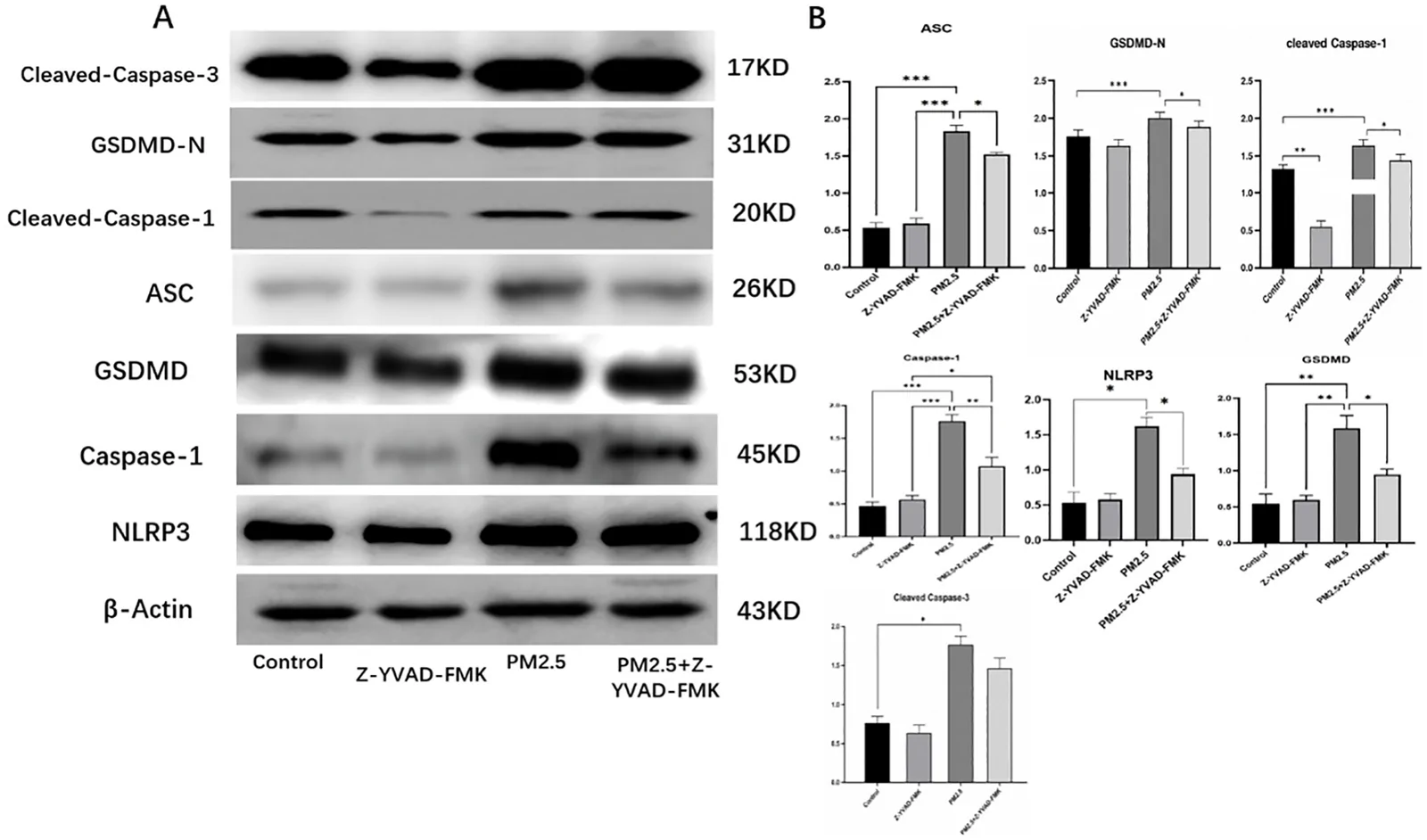
Western blot and quantitative analyses of pyroptosis- and apoptosis-related proteins in rat testicular tissue. (A) Representative bands for cleaved caspase-3, GSDMD-N, cleaved caspase-1, ASC, full-length GSDMD, total caspase-1, NLRP3, and β-actin. Lane order: control, Z-YVAD-FMK, PM2.5, and PM2.5 plus Z-YVAD-FMK. (B) Densitometric quantification normalized to β-actin. Compared with PM2.5 alone, Z-YVAD-FMK co-treatment reduced ASC, GSDMD-N, cleaved caspase-1, total caspase-1, NLRP3, and full-length GSDMD; cleaved caspase-3 did not differ significantly. Horizontal brackets indicate the comparisons shown. *P < 0.05; **P < 0.01; ***P < 0.001.

### 3.2 PM2.5 exposure reduces body and testis weights in rats

As shown in Table 2, neither body weight nor testis weight differed significantly between the Z-YVAD-FMK-only and control groups (P > 0.05). Compared with the control group, the PM2.5 group had lower body weight, testis weight, and testicular organ index (all P < 0.05). Compared with the PM2.5 group, the PM2.5 plus Z-YVAD-FMK group had higher body weight, testis weight, and testicular organ index (all P < 0.05). These findings indicate that PM2.5 exposure was associated with reductions in these measures and that Z-YVAD-FMK treatment was associated with partial recovery.

**Table 2:** Body weight and testicular measurements after PM2.5 exposure.

Group	N	Body weight (g)	Testis weight (g)	Organ index (%)
Control	8	320.7 ± 5.86	1.71 ± 0.10	0.53 ± 0.03
Z-YVAD-FMK	8	322.7 ± 6.66	1.68 ± 0.12	0.52 ± 0.03
PM2.5	8	283.3 ± 10.2*	1.19 ± 0.09*	0.42 ± 0.02*
PM2.5 + Z-YVAD-FMK	8	306.7 ± 2.08#	1.55 ± 0.08#	0.50 ± 0.02#

Note: n = 8 per group. Values are presented as the mean ± standard deviation. *P < 0.05 versus the control group; #P < 0.05 versus the PM2.5 group.

### 3.3 PM2.5 exposure disrupts seminiferous-tubule architecture and germ-cell organization

H&E staining (Fig 2) revealed intact seminiferous tubule architecture and well-organized germ cell layers in both the control and Z-YVAD-FMK-only groups, exhibiting an overall appearance close to normal. In contrast, the PM2.5 group exhibited disrupted seminiferous tubules, enlarged lumina, disorganized germ cells, and vacuolar changes. Notably, these histopathological abnormalities were less pronounced in the PM2.5 plus Z-YVAD-FMK group, suggesting that caspase-1 inhibition may partially mitigate PM2.5-induced testicular injury. These morphological findings provide a phenotypic basis for subsequent molecular analyses. Compared to the control group, rats exposed to 10 mg/kg PM2.5 exhibited a significantly lower sperm count and a significantly higher rate of sperm abnormalities (P < 0.05). Furthermore, compared to the PM2.5 group, the PM2.5 plus Z-YVAD-FMK group demonstrated a higher sperm count and a lower rate of sperm abnormalities (both P < 0.05; Table 3). These findings indicate that PM2.5 exposure reduces sperm numbers and increases morphological abnormalities.

**Table 3:** Changes in sperm parameters after PM2.5 exposure.

Group	N	Sperm count (×10⁶/mL)	Sperm abnormality (%)
Control	8	41.62 ± 3.38	6.31 ± 0.81
Z-YVAD-FMK	8	37.44 ± 3.87	7.16 ± 0.79
PM2.5	8	11.48 ± 4.46*	13.66 ± 1.87*
PM2.5 + Z-YVAD-FMK	8	27.21 ± 3.34#	9.78 ± 0.72#

Note: Values are presented as the mean ± standard deviation for eight rats per group. *P < 0.05 versus the control group; #P < 0.05 versus the PM2.5 group.

### 3.4 PM2.5 exposure alters the expression of pyroptosis- and apoptosis-related proteins in testicular tissue

As shown in Fig 3, PM2.5 exposure increased NLRP3, ASC, total caspase-1, full-length GSDMD, cleaved caspase-1, GSDMD-N, and cleaved caspase-3 relative to the control group (P < 0.05). Compared with the PM2.5 group, the PM2.5 plus Z-YVAD-FMK group showed lower levels of ASC, total caspase-1, full-length GSDMD, NLRP3, cleaved caspase-1, and GSDMD-N (P < 0.05), whereas cleaved caspase-3 did not differ significantly between these two groups. The inhibitor-only group was generally comparable to the control group; however, the plotted comparison for cleaved caspase-1 indicated a lower level in the inhibitor-only group and should be verified against the underlying densitometry before submission. Overall, the protein data suggest that caspase-1 inhibition partially attenuated the PM2.5-associated increase in caspase-1/GSDMD-related signaling, consistent with the reduced histopathological injury observed by H&E staining. qRT-PCR analysis (Fig 7) indicated that, relative to the control group, Hmox1 mRNA expression increased, whereas Stat1, Irf9, and Usp18 mRNA expression decreased in the testes of PM2.5-exposed rats. None of the target genes differed significantly between the Z-YVAD-FMK-only and control groups. Compared with the PM2.5 group, the PM2.5 plus Z-YVAD-FMK group showed lower Hmox1 expression and higher Stat1, Irf9, and Usp18 expression. Because the public dataset was generated from mouse GC-2spd cells and the qRT-PCR experiment used rat testicular tissue, these findings were treated as a cross-system comparison of selected expression patterns rather than direct validation of a common mechanism.

**Fig 4:**
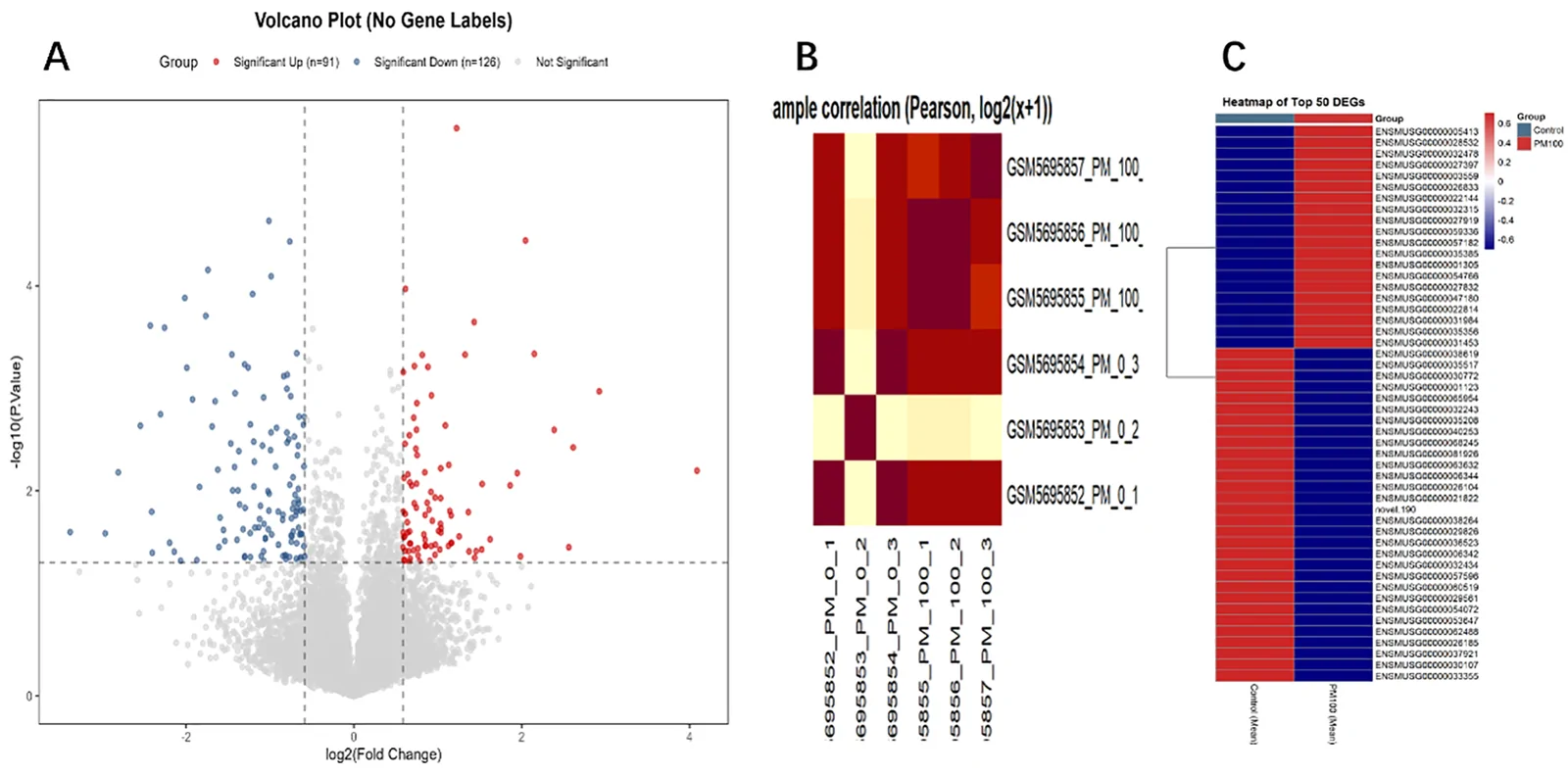
Differential-gene screening and sample expression patterns. This figure shows the differential-expression analysis of PM2.5-treated and control samples in GEO dataset GSE189187. (A) Volcano plot of DEGs; red points denote upregulated genes, blue points denote downregulated genes, and gray points denote genes without significant differential expression. (B) Pearson correlation heatmap. (C) Heatmap of the top-ranked DEGs, showing distinct expression patterns between the PM2.5-treated and control groups and indicating substantial PM2.5-associated transcriptional alterations.

**Fig 5:**
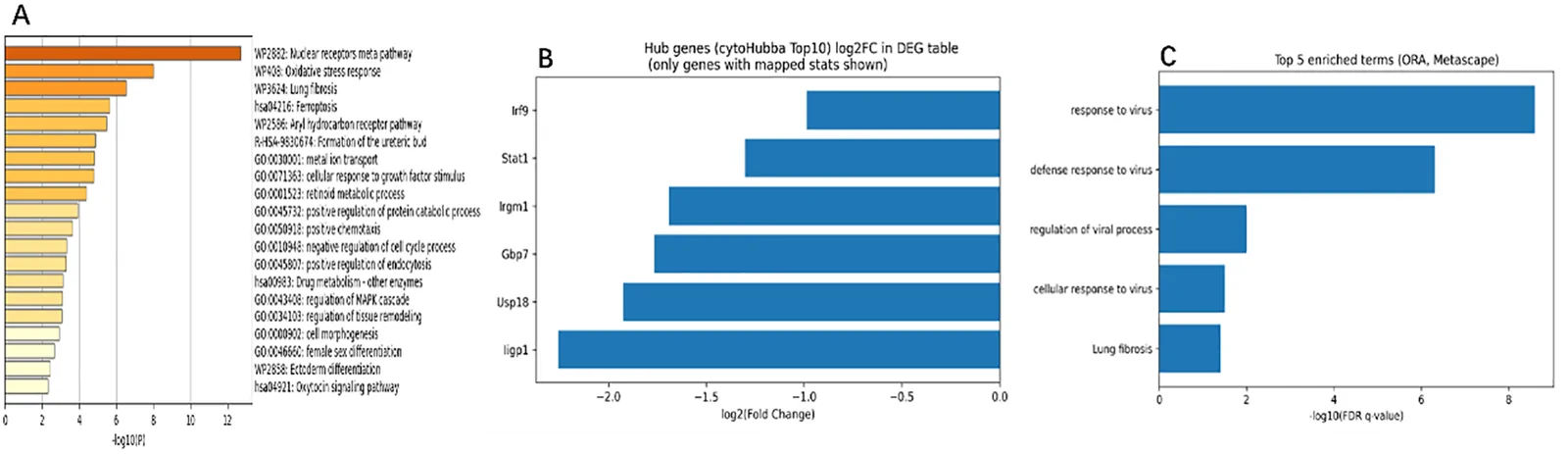
Integrated functional-enrichment analysis of DEGs and expression characteristics of candidate hub genes. (A) Functional-enrichment analysis of the supplied Metascape input, with predominant enrichment in interferon- and antiviral-response terms. (B) log2FC values for mapped cytoHubba-selected genes. (C) The five highest-ranked supplied terms: response to virus, defense response to virus, regulation of viral process, negative regulation of viral process, and regulation of viral life cycle. Lower-ranked terms should be interpreted cautiously after multiple-testing correction.

**Fig 6:**
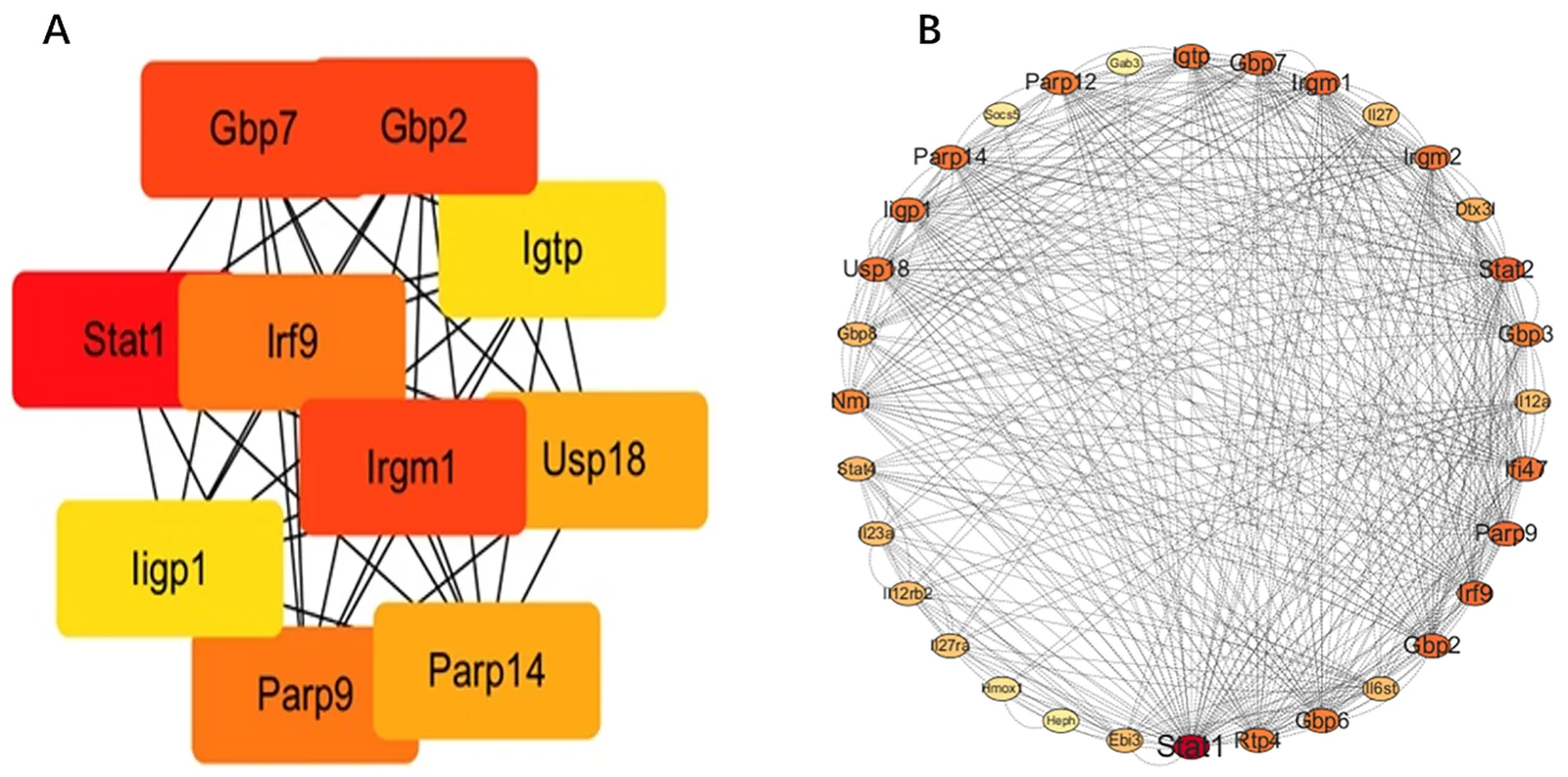
PPI network and hub-gene identification. Exploratory PPI network and hub-gene analysis of an expanded 32-gene pyroptosis/interferon candidate set. (A) Candidate hub genes identified using cytoHubba. (B) STRING/Cytoscape interaction network. The exact node list and DEG-mapping status are provided in S2 File. Because the network was not restricted to the 217 screened DEGs and many nodes did not map to the DEG table, the topology results are hypothesis-generating and should not be interpreted as independent validation of a DEG-derived mechanism.

**Fig 7:**
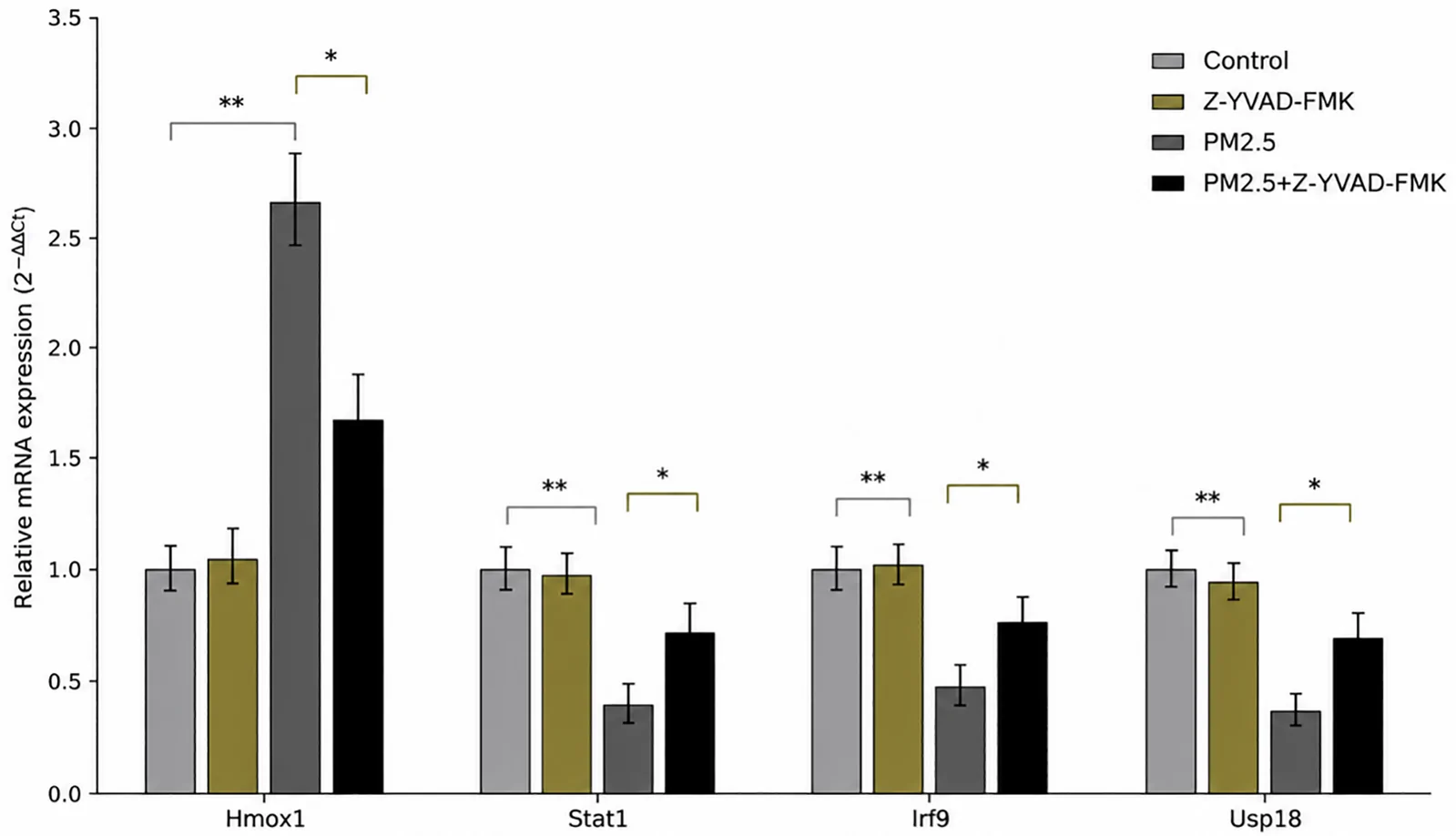
qRT-PCR analysis of candidate genes. Relative mRNA expression of candidate genes in rat testicular tissue across the four experimental groups, as determined by qRT-PCR. Data are presented as the mean ± standard deviation, with three technical replicates per biological sample. Horizontal brackets indicate the specific between-group comparisons. *P < 0.05; **P < 0.01.

### 3.5 PM2.5 exposure is associated with immune-inflammatory and pyroptosis-related pathways

Using the prespecified exploratory thresholds of nominal P < 0.05 and |log2FC| > 0.585 (approximately equivalent to a fold change > 1.5), we identified 217 candidate DEGs in GSE189187 (control, n = 3; PM2.5, n = 3), comprising 91 upregulated and 126 downregulated genes (Figs 4 and 5). Adjusted P values were retained in the supporting output and should be considered when assessing robustness. In the supplied Metascape export, the highest-ranked enrichment terms included response to virus, defense response to virus, regulation of viral process, negative regulation of viral process, and regulation of viral life cycle. The two leading terms remained strongly supported after multiple-testing correction, whereas lower-ranked terms were less robust. Accordingly, the enrichment analysis primarily supports interferon- and antiviral-response remodeling; broader claims regarding specific tissue diseases were removed because they were not supported by the supplied enrichment table.

To focus on the inflammasome/caspase-1/GSDMD-related cascade, the screened DEG set was intersected with a curated pyroptosis-related gene set. Two candidates overlapped: HMOX1, which was upregulated (log2FC = 2.150, nominal P = 4.60 × 10^−4^; adjusted P = 0.294), and STAT1, which was downregulated (log2FC = −1.301, nominal P = 5.83 × 10^−4^; adjusted P = 0.294). Because neither candidate remained significant after false-discovery-rate correction, these findings were treated as hypothesis-generating. An expanded 32-gene pyroptosis/interferon candidate network was analyzed in STRING at a confidence threshold of 0.7 and imported into Cytoscape (Fig 6). The exact node list, module membership, hub ranking, and DEG-mapping status are provided in S2 File. Importantly, this expanded network was not restricted to the 217 screened DEGs, and many nodes did not map back to the DEG table; therefore, MCODE and cytoHubba results were interpreted only as exploratory network context. The top 10 ranked nodes were GBP7, GBP2, STAT1, IRF9, IGTP, IIGP1, IRGM1, USP18, PARP9, and PARP14. qRT-PCR showed increased Hmox1 and decreased Stat1, Irf9, and Usp18 mRNA expression in rat testicular tissue after PM2.5 exposure, with partial reversal after Z-YVAD-FMK treatment (Fig 7). These cross-system expression patterns identify candidates for future protein-level and functional validation but do not establish a conserved causal pathway.

In summary, the public transcriptomic analysis identified candidate signals for PM2.5-associated immune-inflammatory networks. Quantitative reverse transcription polymerase chain reaction (qRT-PCR) further characterized the changes in mRNA expression of Hmox1, Stat1, Irf9, and Usp18 in rat testicular tissue. Histopathological assessments, sperm parameters, and Western blot analyses indicated that PM2.5 exposure induced testicular injury, which was accompanied by enhanced caspase-1/GSDMD-related pyroptotic signaling.

## 4 Discussion

Male infertility is a significant global reproductive health concern, characterized by reduced sperm counts and abnormal morphology as key phenotypic features. In recent decades, global sperm counts have declined, and environmental pollution is considered a potential contributing factor [1]. Epidemiological studies have linked long-term exposure to PM2.5 with an increased risk of male infertility and poorer semen quality [2,4]. Therefore, clarifying the molecular mechanisms by which PM2.5 damages the male reproductive system is crucial. In the present study, intranasal exposure to PM2.5 resulted in a reduction of testis weight and the testicular organ index in rats, disrupted seminiferous tubule architecture, disorganized germ cell layers, and produced vacuolar changes. PM2.5 exposure also led to a decrease in sperm count and an increase in the sperm abnormality rate. These findings indicate that PM2.5 can damage testicular architecture and impair both spermatogenesis and sperm morphology. Previous population-based studies have similarly linked PM2.5 exposure to reduced sperm concentration and abnormal morphology [4,5]. Animal studies have also demonstrated that PM2.5 can cause testicular injury, impair spermatogenesis, and compromise male reproductive function [6,8], which is broadly consistent with our findings. The male reproductive injury induced by PM2.5 likely involves several interacting pathological processes. Previous studies have shown that PM2.5 can damage spermatocytes through mechanisms such as redox imbalance and ferroptosis, and can disrupt testosterone synthesis and testicular endocrine function [6,8]. In the present study, Z-YVAD-FMK partially attenuated testicular injury, the reduction in sperm count, and the increase in sperm abnormalities, suggesting that caspase-1-associated inflammatory signaling may be an important component of PM2.5 reproductive toxicity.

The magnitude of PM2.5-associated reproductive toxicity varies among studies and may depend on factors such as particle source, chemical composition, exposure dose and duration, and route of administration. Environmentally collected particulate matter exhibits significant regional variation in metal and organic pollutant composition, while standard reference materials offer greater batch-to-batch consistency. Different methods of exposure, including intranasal instillation, intratracheal instillation, and inhalation, result in distinct patterns of particle deposition and systemic distribution. Additionally, variations in animal species, age, sperm assessment methods, and sampling time may further contribute to discrepancies among studies. At the molecular level, PM2.5 exposure increases testicular expression of NLRP3, ASC, total caspase-1, full-length GSDMD, cleaved caspase-1, and GSDMD-N, whereas treatment with Z-YVAD-FMK reduces the levels of these proteins. The NLRP3 inflammasome recruits ASC and promotes caspase-1 activation, which subsequently regulates GSDMD-mediated membrane injury and inflammatory responses. GSDMD-mediated pore formation is a critical step in the execution of pyroptosis and the release of inflammatory mediators, and it has been proposed as a therapeutic target in both acute and chronic inflammatory lung diseases. Therefore, our findings support the involvement of NLRP3/caspase-1/GSDMD-related signaling in PM2.5-induced inflammatory injury to the testes.

In addition to caspase-1/GSDMD-related pyroptotic signaling, exposure to PM2.5 increased the levels of cleaved caspase-3 in testicular tissue, suggesting that caspase-3-dependent apoptotic signaling may also contribute to PM2.5-induced testicular injury. Notably, cleaved caspase-3 levels did not differ significantly between the PM2.5 plus Z-YVAD-FMK group and the PM2.5 group, indicating that the inhibition of caspase-1 had a limited effect on apoptosis-related signaling. These findings imply that PM2.5-induced testicular injury may involve multiple forms of regulated cell death, including both pyroptosis and apoptosis, while the protective effect of Z-YVAD-FMK appears to be more closely associated with the attenuation of caspase-1/GSDMD-related signaling. Previous studies on PM2.5-induced pyroptosis have primarily focused on the respiratory system and immune cells. It has been demonstrated that PM2.5 promotes NLRP3 inflammasome activation and IL-1β production in macrophages [12], and enhances pyroptosis-related responses in bronchial epithelial cells through ROS/NF-κB signaling [14]. The analogous changes in NLRP3, caspase-1, and GSDMD observed in rat testicular tissue in the current study suggest that PM2.5-induced inflammasome-related responses may not be confined to the respiratory system, but may also contribute to injury in distal reproductive organs. Unlike pulmonary epithelial cells and macrophages, which are in direct contact with inhaled particles, testicular tissue may be predominantly affected through systemic inflammation, oxidative stress, and alterations in the local immune microenvironment. The blood-testis barrier, the immune privilege of the testes, and reproductive endocrine regulation may impart distinct temporal and quantitative characteristics to testicular inflammatory responses. Furthermore, variations in the sensitivity of germ cells, Sertoli cells, Leydig cells, and immune cells to inflammasome signaling may contribute to the variability observed among experimental models.

Given that Z-YVAD-FMK only partially improved histopathological changes and sperm parameters, it is unlikely that caspase-1-related signaling represents the sole mechanism underlying PM2.5 reproductive toxicity. PM2.5 is also known to induce ferroptosis, oxidative stress, and endocrine disruption [6,7]. The activation of inflammasomes, oxidative stress, and dysregulated iron metabolism may mutually reinforce one another [13]. Therefore, PM2.5-induced testicular injury is more likely to reflect a complex process involving inflammation, metabolic disturbances, and various forms of regulated cell death. Systems biology and bioinformatics approaches can identify key genes, functional modules, and potential regulatory networks from high-dimensional omics data, and have been widely applied to biomarker discovery in respiratory diseases as well as mechanistic studies of hematological disease progression [23–25]. Similar methodologies have been utilized to identify central genes and biological pathways in chronic diseases [26]. Collectively, these studies demonstrate that differential expression, functional enrichment, and protein-protein interaction (PPI) network analyses can provide systems-level mechanistic insights into complex diseases and injuries related to environmental exposure. Our reanalysis of the GSE189187 transcriptomic data from GC-2spd cells revealed that PM2.5-responsive differentially expressed genes (DEGs) were predominantly associated with immune-inflammatory processes, interferon-related responses, oxidative stress, and tissue injury. These findings are consistent with the metabolic and transcriptional alterations reported in previous PM2.5 omics studies [15,16]. Network analysis further identified STAT1, IRF9, members of the GBP family, IRGM1, USP18, PARP9, and PARP14 as candidate hub genes, suggesting that STAT/IRF-related immune networks may play a role in PM2.5-induced cellular stress and inflammation.

HMOX1 contributes to oxidative-stress defense and iron-homeostasis regulation, and its upregulation is biologically compatible with PM2.5-associated redox imbalance [8,13]. STAT1, IRF9, GBP-family proteins, and USP18 are regulators of interferon-associated immunity. In the public dataset, STAT1 and several related genes were altered, suggesting remodeling rather than simple unidirectional activation of the STAT/IRF network. Although STAT1 is required to establish IFN-γ-induced transcriptional memory, it is not required to maintain that state [21]; exposure duration, cell type, and negative-feedback regulation may therefore influence expression direction. The public transcriptomic analysis, rat-tissue qRT-PCR measurements, and animal phenotypes provide complementary but non-equivalent evidence. Species, sample type, and exposure-system differences preclude treating qRT-PCR in rat testes as direct validation of the GC-2spd network. Together, the findings support an association between PM2.5 exposure, oxidative stress, immune-inflammatory remodeling, abnormal sperm parameters, and increased caspase-1/GSDMD-related protein expression, while the candidate network requires independent validation.

In summary, intranasal exposure to PM2.5 disrupted testicular architecture, reduced sperm count, increased the rate of sperm abnormalities, and enhanced NLRP3/caspase-1/GSDMD-related signaling in rats. These changes were partially attenuated by Z-YVAD-FMK. When considered alongside previous evidence, our findings support a role for caspase-1-associated inflammatory signaling in PM2.5-induced male reproductive injury. Furthermore, they suggest that oxidative stress and the remodeling of STAT/IRF-related immune networks may jointly contribute to its molecular basis.

## 5 Conclusions

Intranasal PM2.5 exposure induced testicular histopathological injury, reduced sperm count, increased sperm abnormalities, and enhanced NLRP3/caspase-1/GSDMD-related signaling in rats. Z-YVAD-FMK partially mitigated these changes, whereas cleaved caspase-3 was not significantly reduced relative to the PM2.5 group. Public transcriptomic analysis suggested interferon- and immune-inflammatory transcriptional remodeling in PM2.5-treated GC-2spd cells. Rat-tissue qRT-PCR provided a cross-system comparison for Hmox1, Stat1, Irf9, and Usp18, but did not establish functional validation of the candidate network. The STAT/IRF-GBP-IRGM-USP18/PARP network should therefore be regarded as hypothesis-generating. Additional protein-level measurements, cellular localization studies, and targeted functional interventions are required to establish the roles of these candidates in PM2.5-induced testicular injury.

## Supporting information

S1 File Original uncropped Western blot source images and annotated source-image index. (ZIP)

S2 File Bioinformatics analysis archive for GSE189187, including differential-expression and enrichment outputs, STRING/PPI network files, MCODE modules, cytoHubba hub-gene results, and DEG-mapping materials. (ZIP)

S1 Fig Original H&E staining images. (PDF)
