# Supplementary material for: Nasal exposure to PM2.5 induces testicular injury and activation of pyroptosis-related signaling in rats: Inhibitor intervention and public transcriptomic analysis
Source: PLoS One. 2026 Sep 15;21(9):e0357773. doi: 10.1371/journal.pone.0357773 (PMC13577368; doi:10.1371/journal.pone.0357773)
Supplement: Supplementary file 1 [file pone.0357773.s003.pdf]

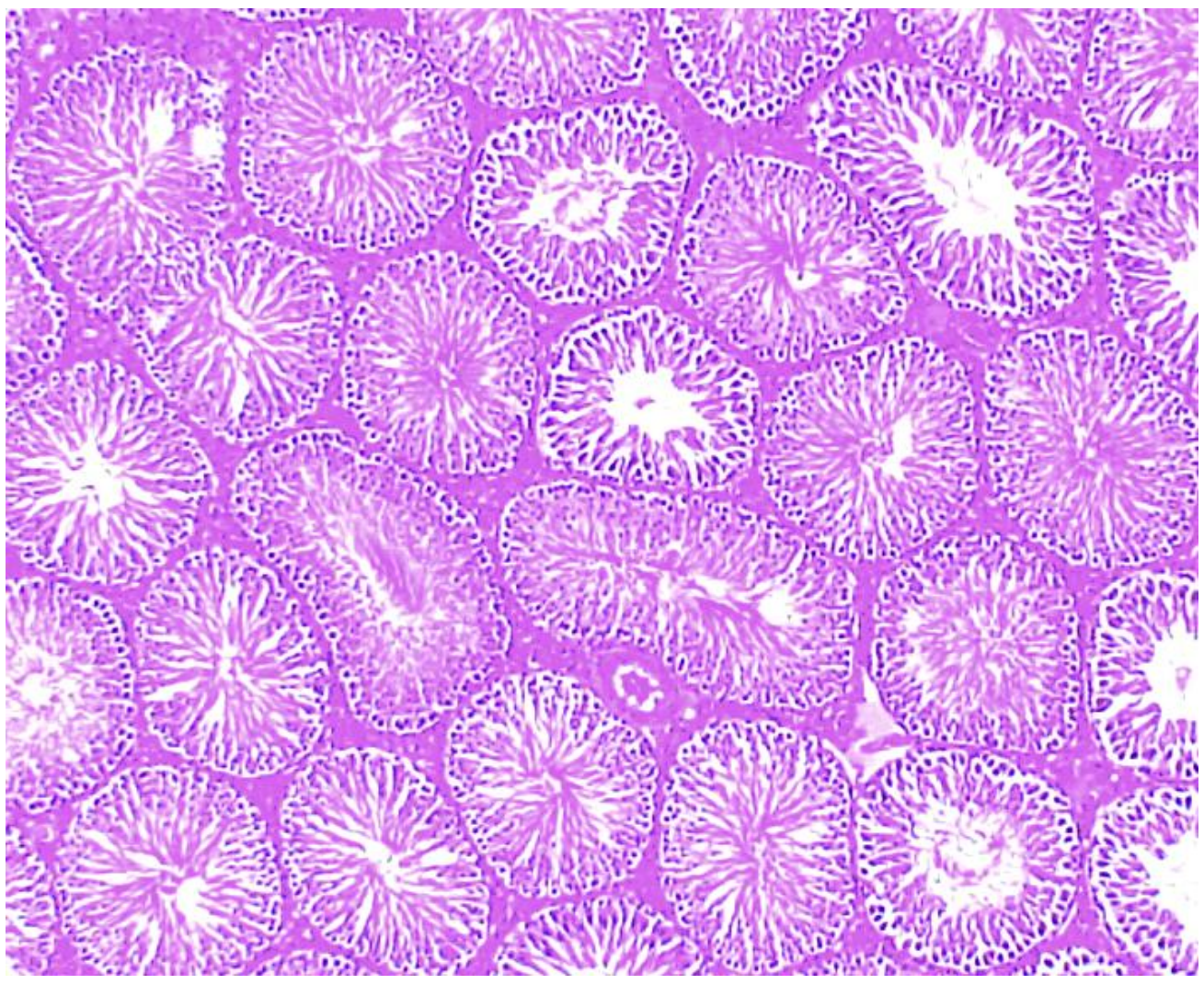

Control

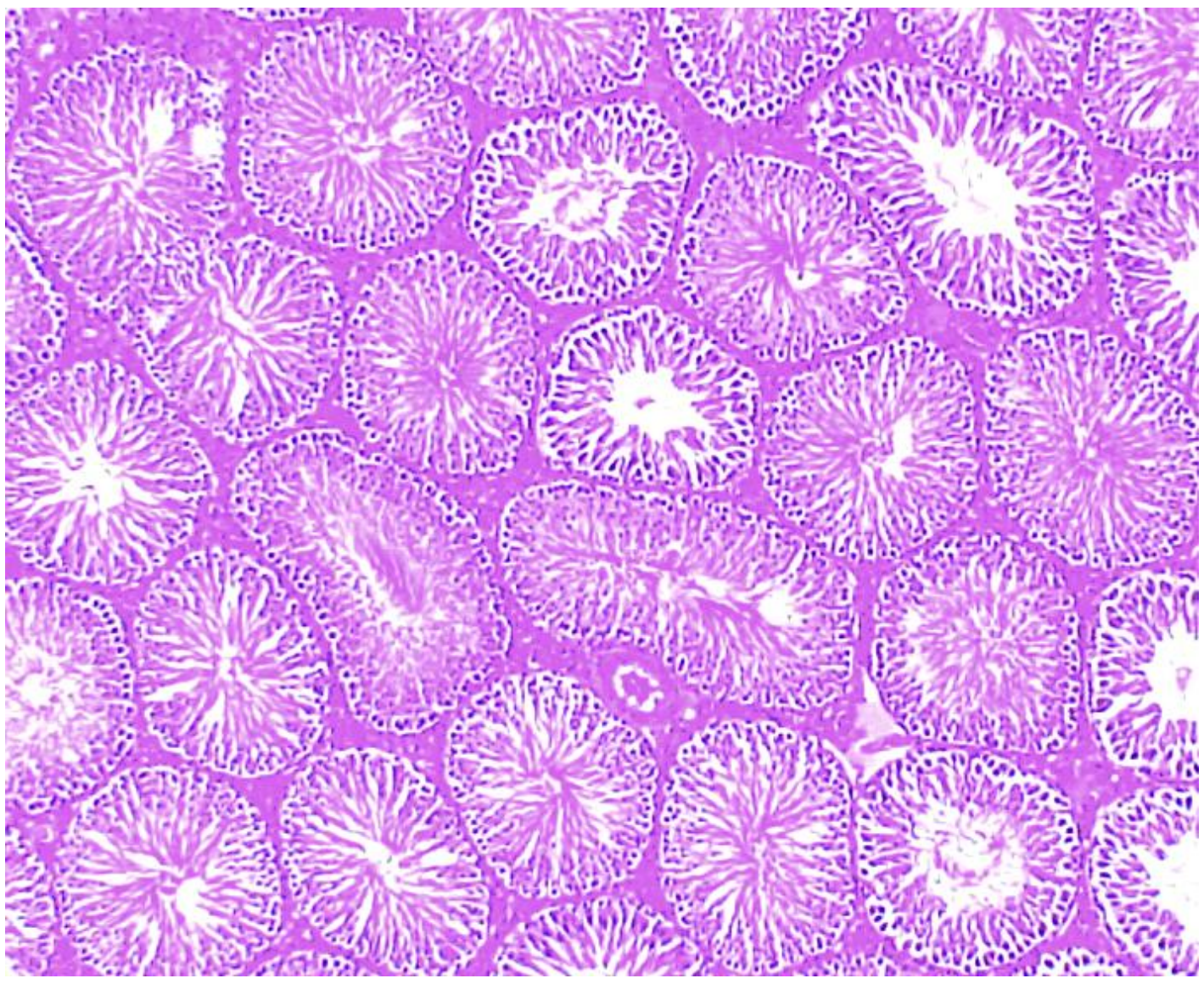

Z-YVAD-FMK

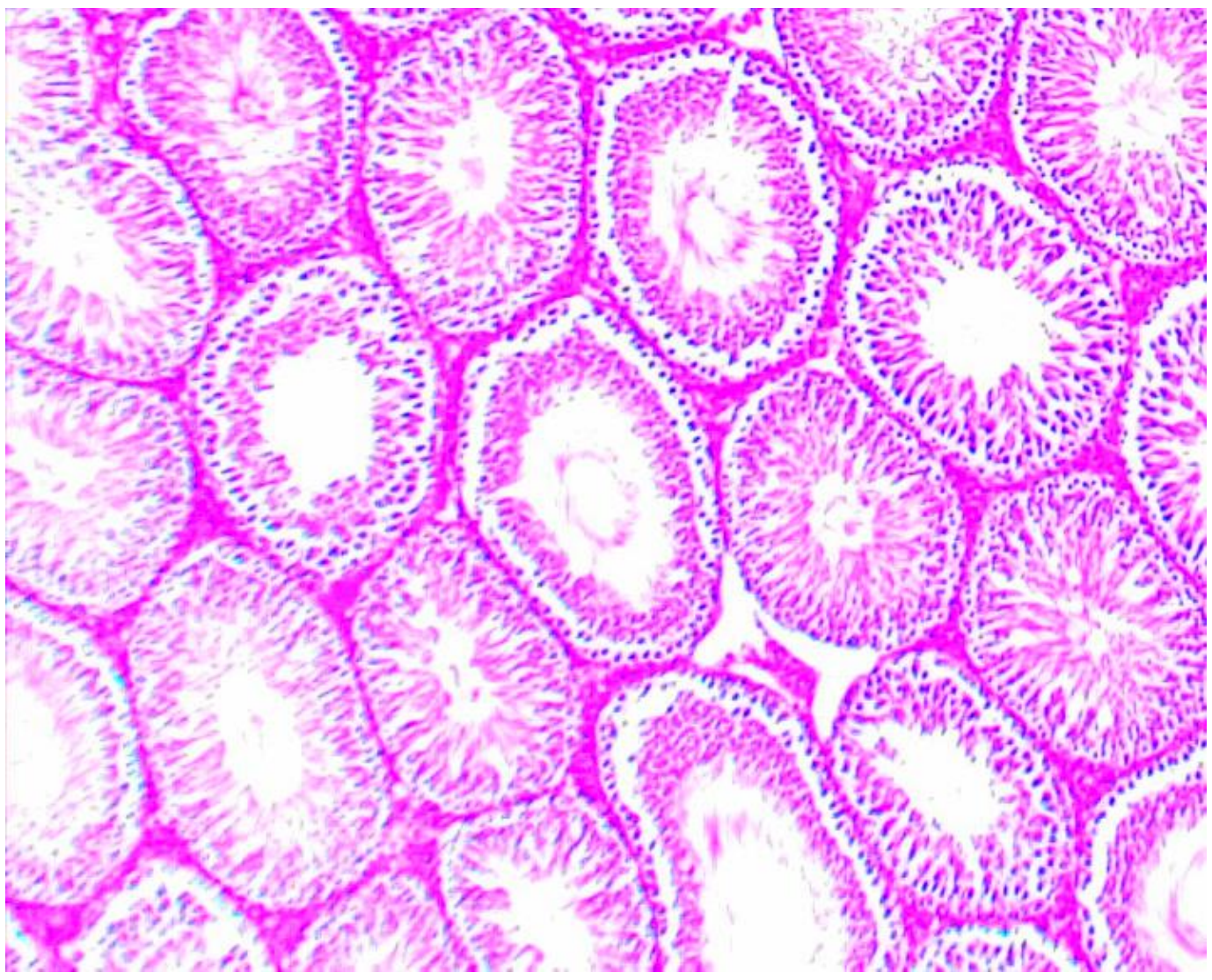

PM2.5

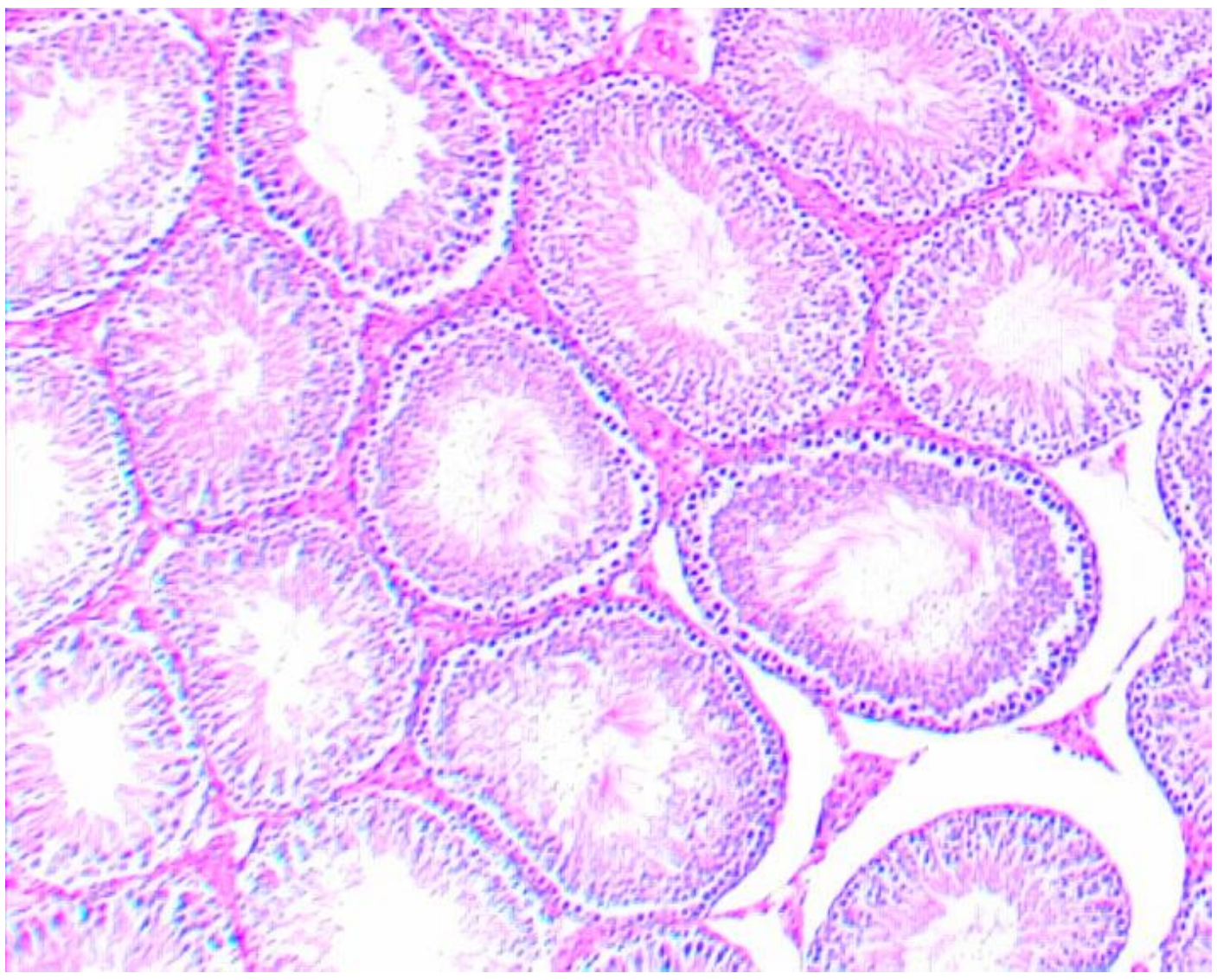

PM2.5+Z-YVAD-FMK
