# Supplementary material for: Nasal exposure to PM2.5 induces testicular injury and activation of pyroptosis-related signaling in rats: Inhibitor intervention and public transcriptomic analysis
Source: PLoS One. 2026 Sep 15;21(9):e0357773. doi: 10.1371/journal.pone.0357773 (PMC13577368; doi:10.1371/journal.pone.0357773)
Supplement: Supplementary file 2 [file pone.0357773.s001.zip › S1_raw_images.docx]

**S1_raw_images. Original Uncropped Western Blot Images**
This file contains the original uncropped and minimally adjusted Western blot images corresponding to Fig 3A. The target proteins include ASC, GSDMD, Caspase-1, NLRP3, and the internal reference protein β-Actin. Unless otherwise indicated, the lane order is as follows: Lane 1, Control group; Lane 2, Z-YVAD-FMK group; Lane 3, PM2.5 group; Lane 4, PM2.5 + Z-YVAD-FMK group, arranged from left to right. Images were captured using a Tanon imaging system。

**1.Protein: ASC
2.Corresponding figure: Corresponding to Fig 3A; used for densitometric analysis in Fig 3B.
3.Lane order: Lanes 1–4 are arranged from left to right.
4.Sample identity: Lane 1, Control group; Lane 2, Z-YVAD-FMK group; Lane 3, PM2.5 group; Lane 4, PM2.5 + Z-YVAD-FMK group.
5.Molecular weight: 22–26 kDa.
6.Detection method: Chemiluminescence imaging using a Tanon imaging system.
7.Image type: Original uncropped Western blot image.**

**1.Protein: β-Actin
2.Corresponding figure: Corresponding to Fig 3A; used as the internal reference protein for densitometric normalization.
3.Lane order: Lanes 1–4 are arranged from left to right.
4.Sample identity: Lane 1, Control group; Lane 2, Z-YVAD-FMK group; Lane 3, PM2.5 group; Lane 4, PM2.5 + Z-YVAD-FMK group.
5.Molecular weight: 42–43 kDa.
6.Detection method: Chemiluminescence imaging using a Tanon imaging system.
7.Image type: Original uncropped Western blot image.**

**1.Protein: Caspase-1
2.Corresponding figure: Corresponding to Fig 3A; used for densitometric analysis in Fig 3D.
3.Lane order: Lanes 1–4 are arranged from left to right.
4.Sample identity: Lane 1, Control group; Lane 2, Z-YVAD-FMK group; Lane 3, PM2.5 group; Lane 4, PM2.5 + Z-YVAD-FMK group.
5.Molecular weight: 45 kDa.
6.Detection method: Chemiluminescence imaging using a Tanon imaging system.
7.Image type: Original uncropped Western blot image.**

**1.Protein: GSDMD
2.Corresponding figure: Corresponding to Fig 3A; used for densitometric analysis in Fig 3C.
3.Lane order: Lanes 1–4 are arranged from left to right.
4.Sample identity: Lane 1, Control group; Lane 2, Z-YVAD-FMK group; Lane 3, PM2.5 group; Lane 4, PM2.5 + Z-YVAD-FMK group.
5.Molecular weight: 53 kDa.
6.Detection method: Chemiluminescence imaging using a Tanon imaging system.
7.Image type: Original uncropped Western blot image.**

**1.Protein: NLRP3
2.Corresponding figure: Corresponding to Fig 3A; used for densitometric analysis in Fig 3E.
3.Lane order: Lanes 1–4 are arranged from left to right. The leftmost lane represents the molecular weight marker.
4.Sample identity: Lane 1, Control group; Lane 2, Z-YVAD-FMK group; Lane 3, PM2.5 group; Lane 4, PM2.5 + Z-YVAD-FMK group.
5.Molecular weight: 118 kDa.
6.Detection method: Chemiluminescence imaging using a Tanon imaging system.
7.Image type: Original uncropped Western blot image**
