# Supplementary material for: Nasal exposure to PM2.5 induces testicular injury and activation of pyroptosis-related signaling in rats: Inhibitor intervention and public transcriptomic analysis
Source: PLoS One. 2026 Sep 15;21(9):e0357773. doi: 10.1371/journal.pone.0357773 (PMC13577368; doi:10.1371/journal.pone.0357773)

Sample correlation (Pearson, log2(x+1))

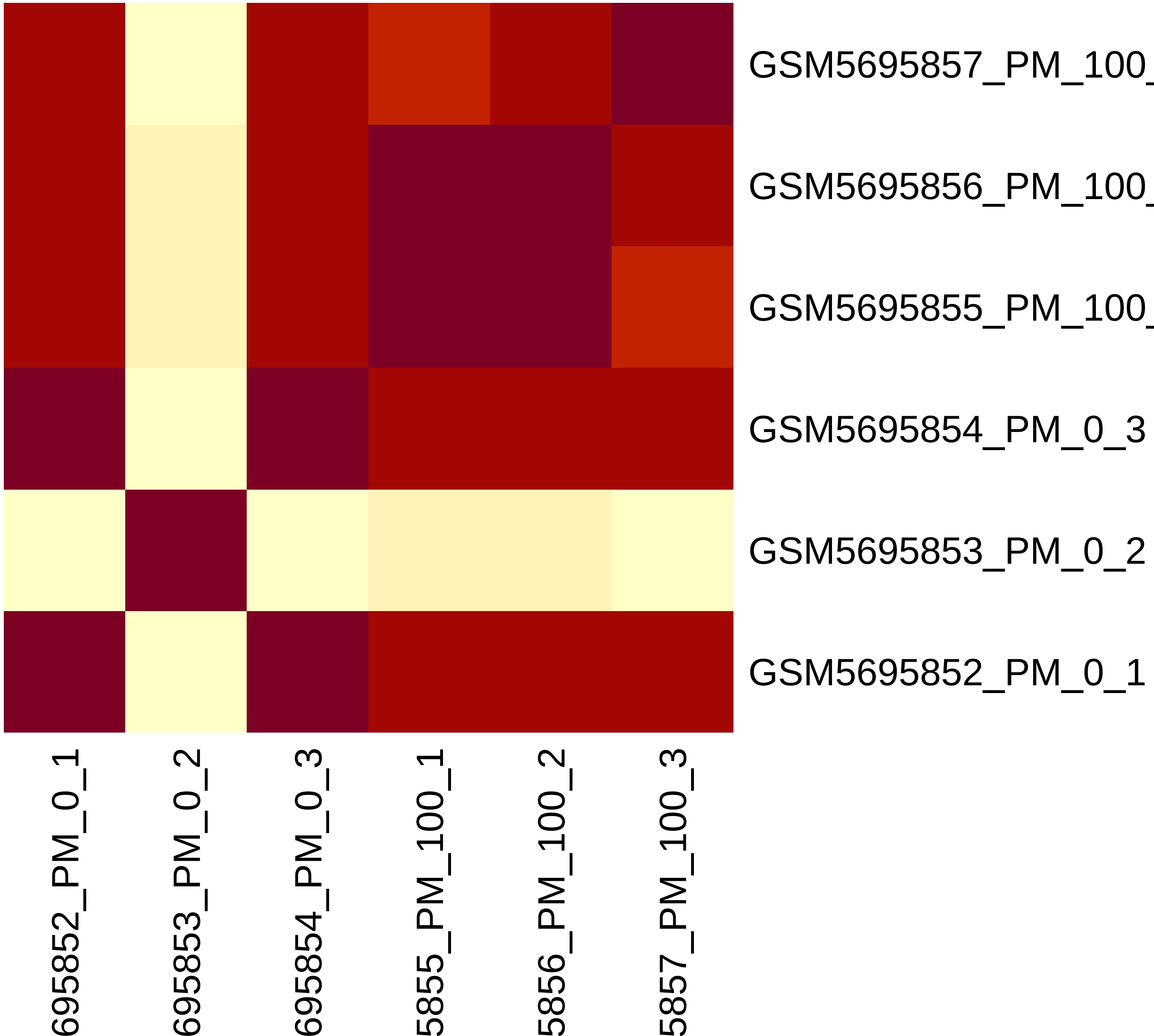

Supplement: Supplementary file 3 [file pone.0357773.s002.zip › MCODEand CytoHubba2026-4-26/A3_CorrelationHeatmap.pdf]
