# Supplementary material for: Nasal exposure to PM2.5 induces testicular injury and activation of pyroptosis-related signaling in rats: Inhibitor intervention and public transcriptomic analysis
Source: PLoS One. 2026 Sep 15;21(9):e0357773. doi: 10.1371/journal.pone.0357773 (PMC13577368; doi:10.1371/journal.pone.0357773)

Top50 DEGs Heatmap (6 samples, row z−score, method=P)

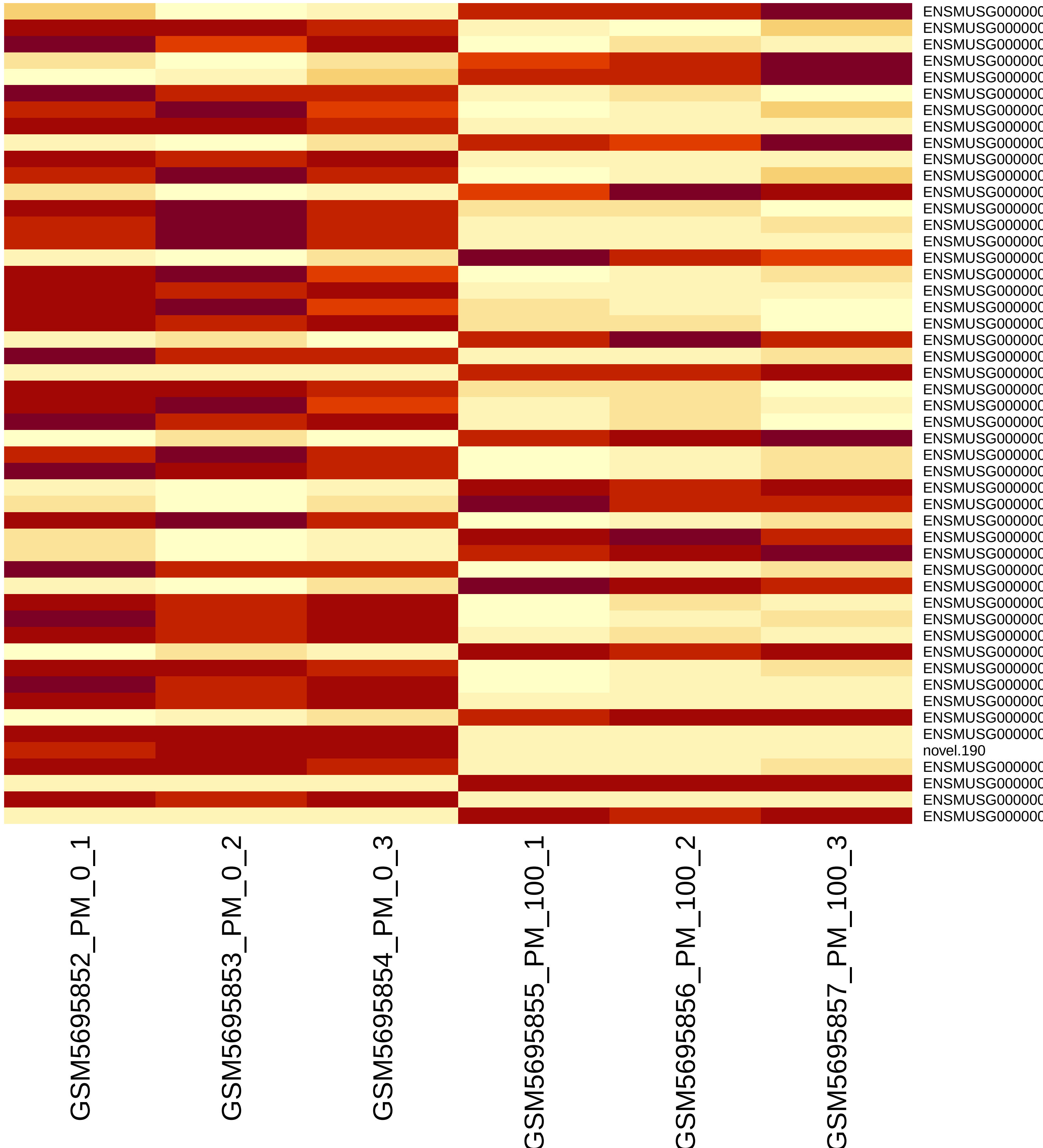

Supplement: Supplementary file 3 [file pone.0357773.s002.zip › MCODEand CytoHubba2026-4-26/Heatmap_Top50_6samples_P.pdf]
